# Gaseous Air Pollutants and Lung Function in Fibrotic Interstitial Lung Disease (fILD): Evaluation of Different Spatial Analysis Approaches

**DOI:** 10.1021/acs.est.4c11275

**Published:** 2025-03-22

**Authors:** Shuangjia Xue, Matthew J. Broerman, Gillian C. Goobie, Daniel J. Kass, James P. Fabisiak, Sally E. Wenzel, Seyed Mehdi Nouraie

**Affiliations:** † Department of Environmental and Occupational Health, Graduate School of Public Health, University of Pittsburgh, Pittsburgh, Pennsylvania 15261, United States; ‡ Division of Pulmonary, Allergy, Critical Care, and Sleep Medicine, Department of Medicine, University of Pittsburgh, Pittsburgh, Pennsylvania 15213, United States; § Division of Respiratory Medicine, Department of Medicine, University of British Columbia, Vancouver, British Columbia V5Z 1M9, Canada; ∥ Centre for Heart Lung Innovation, St. Paul’s Hospital, University of British Columbia, Vancouver, British Columbia V5Z 1M9, Canada; ⊥ Simmons Center for Interstitial Lung Disease, Division of Pulmonary, Allergy and Critical Care Medicine, Department of Medicine, University of Pittsburgh, Pittsburgh, Pennsylvania 15213, United States

**Keywords:** gaseous pollutants, interstitial lung disease, geospatial models, kriging, cross-validation

## Abstract

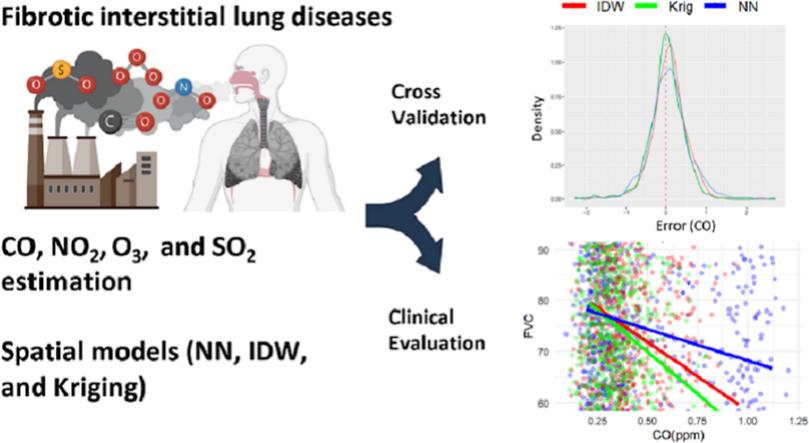

Gaseous pollutants such as CO, NO_2_, O_3_, and SO_2_ are linked to adverse clinical outcomes in patients with fibrotic interstitial lung diseases (fILDs), particularly idiopathic pulmonary fibrosis. However, the effect of various exposure estimation methods on these findings remains unclear. This study aims to evaluate three spatial approaches—nearest neighbor (NN), inverse distance weighting (IDW), and Kriging—for estimating gaseous pollutant exposures and to assess how these methods affect health outcome estimates in fILD patients. A 10-fold cross-validation showed that Kriging had the lowest prediction error compared to NN and IDW, with RMSE for CO = 0.43 ppm (11%), O_3_ = 5.9 ppb (14%), SO_2_ = 2.7 ppb (12%), and NO_2_ = 7.6 ppb (9%), respectively. Kriging also excelled over other methods across wide spatial and temporal ranges, showing the highest spatial *R*^2^ for CO and O_3_ and the highest temporal R^2^ for SO_2_ and NO_2_. In a large cohort of patients with fILD, higher levels of CO, SO_2_, and NO_2_ exposure were associated with lower pulmonary function. The magnitude of association and its precision were higher in SO_2_ and CO estimated by the Kriging method. This study underscores Kriging as a robust method for estimating gaseous pollutant levels and offers valuable insights for future epidemiological studies.

## Abstract 2

Evaluating spatial methods for estimating gaseous pollutant exposures and their impact on health outcomes in fibrotic interstitial lung disease.

## Introduction

Fibrotic interstitial lung diseases (fILDs) make up a heterogeneous group of conditions characterized by progressive pulmonary fibrosis, high morbidity, and early mortality. For people with the most severe form of fILD, namely, idiopathic pulmonary fibrosis (IPF), median life expectancy is around 3–5 years after diagnosis,^2^ and personal and environmental risk factors can further exacerbate this.^1,3−9^ Our group has demonstrated in a large and diverse cohort of patients with diverse forms of fILD that increased exposures to PM ≤ 2.5 μm in diameter (PM_2.5_) and its human-derived constituents are associated with worse lung function and shorter transplant-free survival.^10^ What remains unclear is the impact of gaseous air pollutants on clinical outcomes in patients with diverse forms of fILD and the appropriate exposure methodology for studying these effects.^7,11^

The Environmental Protection Agency (EPA) regulates the gaseous pollutants carbon monoxide (CO), nitrogen dioxide (NO_2_), ozone (O_3_), and sulfur dioxide (SO_2_). CO, mainly sourced from vehicle and industrial emissions, impairs hemoglobin’s oxygen uptake and adversely impacts cardiovascular and respiratory functions.^12^ NO_2_, formed from combustion, reacts with air moisture to create nitric acid, leading to pulmonary inflammation.^3−5,13^ O_3_, a secondary pollutant, forms when nitrogen oxides and volatile organic compounds react under UV radiation.^14^ This reactive oxygen species can cause coughing, shortness of breath, airway irritation, inflammation, and reduced lung function.^4,15^ SO_2_, mainly from fossil fuel combustion, reacts with water to form sulfuric acid, irritating the respiratory tract.^16,17^ Different pollutants impact lung function through distinct mechanisms; for instance, SO_2_ primarily affects lung volume,^18^ while NO_2_ interacts more directly with the alveolar-capillary membrane, affecting lung diffusing capacity.^19^ These variations highlight the need to examine pollutants individually rather than grouping them under ″air pollution″ as a whole.

While there is a growing body of evidence linking particulate and gaseous pollutant exposures with adverse outcomes in patients with fILD, there is a lack of clarity on the appropriate methodological approach for pollution exposure assignment in clinical cohorts. Epidemiological studies require accessible air pollution measurements covering large geographic areas, refined to repeated discrete time points and available at a low cost. Satellite measurement is an option for estimating PM_2.5_ exposures but is not ideal for gaseous pollutants. Only NO_2_ data are available from NASA’s air quality monitoring program since 2005, and these data lack fine temporal resolution.^20^ Epidemiological studies commonly rely on ground-based monitors and interpolation,^21^ yet there remains a need for a transparent and universally accepted modeling framework for extrapolation to areas lacking monitoring coverage. Inverse distance weighting (IDW) and nearest neighbor (NN) modeling are commonly used statistical approaches for air pollution exposure estimation in clinical populations.^22,23^ These approaches are low in computation cost and allow applications to be used over larger areas. A disadvantage of statistical models is their lack of temporal coverage. Outliers and short-term changes influence the temporal validity of these exposure estimations.^22^ Kriging is a linear unbiased estimator for predictions at unsampled locations, which explicitly considers spatial autocorrelation and dependencies.^24^ However, the quality of Kriging predictions can be sensitive to the chosen variogram model.^25,26^

Patients with fILD are often older with significant comorbidities, making direct measurements logistically difficult and cost-prohibitive. Even small inaccuracies in exposure estimation could attenuate or obscure exposure–response relationships due to the disease’s heterogeneous progression and sensitivity to environmental factors. The long-term duration of this study creates a unique opportunity to assess the robustness of each of these three methods in this study population. To date, no studies have compared different methodologies for estimating exposures to gaseous air pollutants in patients with fILD, so it remains unclear how these alternative methods may influence health effect estimates. This study aimed to build an analytical pipeline to interpolate air pollution measures in epidemiological studies using NN, IDW, and Kriging techniques. Our goal was to measure the validity of these three approaches for estimating patient exposures to gaseous pollutants over a long-term period and a large area. By directly comparing these methods, our study aims to optimize exposure estimation for this vulnerable population, ensuring better alignment of exposure assessment with health outcomes. We applied these exposure interpolations to patients with fILD to identify a suitable exposure model for assessing the association between gaseous pollutants and clinical outcomes in this population.

## Methods

### Study Population

Adult patients (≥18 years old) fulfilling diagnostic criteria for fILD including IPF, fibrotic hypersensitivity pneumonitis (fHP), connective tissue disease-ILD (CTD-ILD), non-IPF idiopathic interstitial pneumonia (IIP), pneumoconiosis, and unclassifiable ILD were prospectively enrolled in the University of Pittsburgh Dorothy P. and Richard P. Simmons Center for Interstitial Lung Disease Registry in Pittsburgh, Pennsylvania, between 2000 and 2021. Patients’ residential location (from the Northeastern US) at the time of registry enrollment was collected (using the full address), alongside clinical and demographic data. Ethics approval (STUDY20050209) was obtained from the University of Pittsburgh, and patients provided written consent for registry enrollment. Baseline FVC and DLCO were defined as the first tests performed within 6 months of enrollment. FVC and DLCO are two important markers of ILD progression and treatment response that are used to calculate severity score (GAP), which is strongly associated with prognosis among patients with ILD.^27−29^ Age and sex were already adjusted for in the percent predicted values of DLCO and FVC that are used in this study. Smoking history was classified based on self-reported data. We categorized participants as ″never”, ”former”, or ″current″ smokers. Other demographic details of the study population are available from our prior publication.^10^

### Air Pollution Estimation

The ambient air concentrations of sulfur dioxide (SO_2_), carbon monoxide (CO), ozone (O_3_), and nitrogen dioxide (NO_2_) were obtained from the United States (US) EPA Air Data repository.^30^ Concentration data for SO_2_ (measured in parts per billion (ppb), from 1669 US-wide monitors), O_3_ (measured in ppb, from 2515 US-wide monitors), NO_2_ (measured in ppb, from 1210 US-wide monitors), and CO (measured in parts per million (ppm), from 1196 US-wide monitors) were available from January 1990 to April 2022. These measurements encompass a broad geographic range across the US, with higher monitor density observed along coastal regions (Figure S1). Three interpolation methods, including matching to the NN, IDW, and Kriging, were applied to estimate pollutant exposures at patients’ residential locations.^31^ The NN method assigned exposure based only on the single monitor closest to the participant’s residence. IDW calculated the patients’ exposure by averaging the weighted values at nearby monitors (up to 5 neighbors) within 20 km, where the weight was a function of the inverse distance between the patient and the monitor.^7^ We used λ = 1/*d*_*i*_^2^ as a weighting factor for monitor *i*, where *d*_*i*_ was the distance between monitor i and the point to be predicted, and 2 was the power parameter. Kriging reflected a weighted combination of monitor values, but unlike IDW, Kriging took spatial correlation into consideration, which was reflected by the distance and directions between monitors. Kriging was applied in three steps: (1) a function was fit to the empirical variogram, which is the degree of dissimilarity between two observations separated by a given distance; (2) three frequently applied covariance models (spherical, exponential, and Gaussian) were fit with the best-fitting model identified based on corresponding nuggets, sills, and ranges based on the variogram, with parameters listed in Table S1; and (3) daily concentrations of gaseous air pollutants were estimated for each study subject. Patient exposure levels were calculated as averages over 1, 6, 12, and 24 months surrounding the pulmonary function test (PFT) date. For example, the 1 year average represents exposure levels measured 6 months before and 6 months after the PFT date.

### Cross-Validation

10-Fold cross-validation was applied to compare the internal validity of the three interpolation models. This was performed by splitting all monitoring sites into 10 splits, followed by training with 90% of the data and predicting SO_2_, CO, O_3_, and NO_2_ at the remaining 10% of monitoring sites. The same process was repeated 10 times. Predictions were assembled from all ten splits for every month from January 1990 to April 2022, with spatial and temporal *R*^2^s calculated between predicted and monitored pollution levels at each monitoring site.^32^ Spatial *R*^2^ accounts for the interdependence of observations based on geographic proximity, reflecting spatial autocorrelation. Temporal *R*^2^ measures the dependencies and trends in observations over time, indicating how past observations influence current states. Air pollution estimation residuals were computed as predicted values (_i) minus observed values (y_i) across interpolation methods. The Root Mean Square Error (RMSE = √(∑(e_i^^^2)/*n*), where e_i = *y*_i–ŷ_i) was used to estimate the total magnitude of errors for each pollutant, across monitoring stations and for all periods. The proportional RMSE was calculated by dividing the range (maximum–minimum) of each pollutant. These RMSE results provided insights into the precision of each modeling approach, while the percentages offered context by comparing errors to pollutant concentration scales. For example, if the real measurement of a pollutant at monitor A is 3.0 ppb, and our five estimations are 2.9, 2.8, 3.1, 3.3, and 2.7, we calculate the RMSE to be 0.22 ppb, based on √(((3.0–2.9)^2^ + (3.0–2.8)^2^ + (3.0–3.1)^2^ + (3.0–3.3)^2^ + (3.0–2.7)^2^)/5), with the proportional RMSE of 0.22/(3.3–2.7) = 0.35.

The overall temporal *R*^2^ was computed by conducting a regression analysis of the Δ*m*easurement against the Δ*p*redicted. Here, the Δ*m*easurement represents the variation between the actual pollutant levels at location *i* at time *t* and the annual mean pollutant levels at that same location. Similarly, Δ*p*redicted corresponds to the analogous variation for the predicted pollutant values generated by the model.^33^ The overall spatial *R*^2^, on the other hand, was determined through a regression analysis that involved the annual mean pollutant levels at location *i* regressed against the annual mean predicted pollutant levels at the same location. These statistics provide a comprehensive view of the temporal *R*^2^ performance across different pollutants and modeling methods, offering insights into their respective predictive capabilities.

### Statistical Analysis

Associations of air pollutants with baseline percent predicted forced vital capacity (FVC %) and the diffusion capacity of the lung for carbon monoxide (DLCO) were assessed with linear regression. FVC was converted to age- and sex-based percent predicted using race-neutral GLI global (2022) equations built in ‘rspiro’ package in *R*, which applied inverse probability weights so that each racial and ethnic group contribute equally to the predicted values. We calculated three estimates for each association: one unadjusted, one adjusted for smoking history, and one adjusted for smoking history and ILD type (IPF). For comparing different exposure time windows, we converted exposure (1, 6, 12, and 24 months) to z-scores (standardizing them) and then performed linear regression. The resulting coefficient indicating the relationship in standard deviation units was summarized and is presented in the line charts. All analyses were performed with *R*, version 4.2.2.^34,35^

## Results

### Baseline Characterization

The baseline characteristics of 1424 patients with fILD included are summarized in Table 1. The median age at registry enrollment was 66 years (interquartile range [IQR]: 58–73). Among the participants, 795 (56%) were male and 1307 (91.8%) identified as White. The most common diagnosis was IPF in 717 patients (50%), followed by connective tissue disease-ILD (CTD-ILD) in 300 (20%). Median race-neutral FVC percent predicted was 74 (IQR: 59–90) at enrollment, and the median DLCO percent predicted was 49 (IQR: 37–63).

**Table 1:** Demographic Characteristics of Study Population

characteristic	all patients, *N* = 1424
age, median (IQR)	66 (58 to 73)
sex, *n* (%)	
male	795 (56)
race, *n* (%)	
asian	6 (0.4)
black	57 (4.0)
indigenous	3 (0.2)
white	1307 (91.8)
unknown	52 (3.7)
diagnosis	
idiopathic pulmonary fibrosis (IPF)	717 (50.3)
connective tissue disease-ILD (CTD-ILD)	300 (20.1)
fibrotic hypersensitivity pneumonitis (fHP)	55 (3.9)
pneumoconiosis	26 (1.8)
non-IPF idiopathic interstitial pneumonia	68 (4.8)
other fibrotic interstitial lung disease (fILD)	50 (3.5)
unclassifiable or not yet diagnosed	209 (14.7)
smoke history (always/former/never)	38/665/412
baseline FVC % predicted, median (IQR)	74 (59 to 90)
baseline DLCO % predicted, median (IQR)	49 (37 to 63)

### Summary Statistics of Gaseous Pollutants

**Figure 1:**
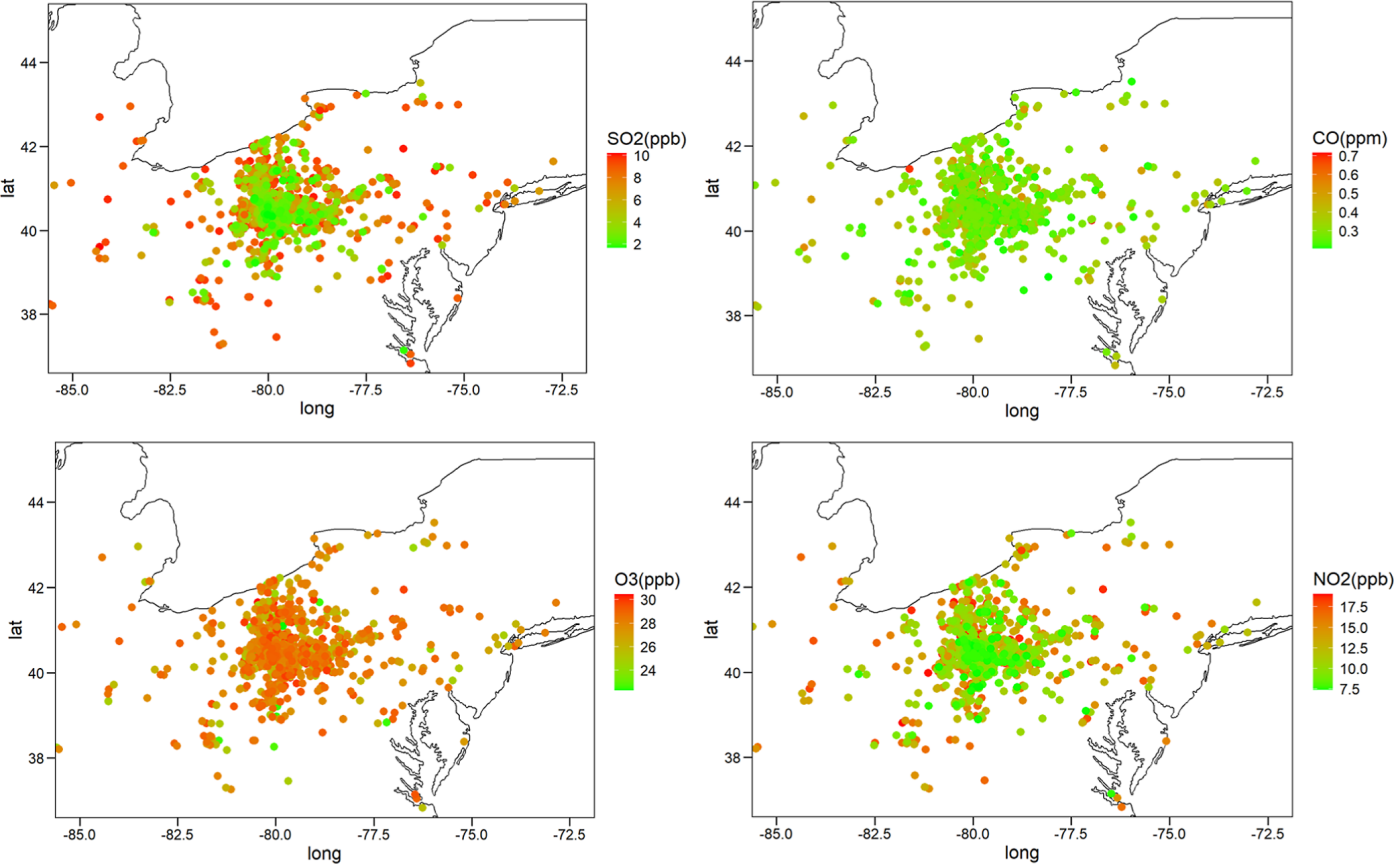
Levels of gaseous pollutants (12 months average before the date of enrollment) at patients’ locations based on Kriging (IDW and NN look similar on the national scale map). The EPA limit for SO_2_ is 75 ppb for 1 h and 500 ppb for 3 h. For CO, the EPA limit is 35 ppm for 1 h and 9 ppm for 8 h. For O_3_, the EPA limit is 70 ppb for 8 h, and for NO_2_, the EPA limit is 100 ppb for 1 h and 53 ppb for 1 year.^58^

The exposure levels of SO_2_, CO, O_3_, and NO_2_ at patients’ locations were evaluated through three different interpolation approaches (NN, IDW, and Kriging). The average exposure was calculated 1 year before each patient’s registry enrollment date. The 1 year pre-enrollment median (IQR) for SO_2_ was 7.20 (5.27–8.57), 7.66 (5.19–8.90), and 7.35 (4.46–9.01) ppb for NN, IDW, and Kriging, respectively. Following the same order, the median (IQR) for CO was 0.31 (0.26–0.57), 0.34 (0.30–0.44), and 0.31 (0.28–0.39) ppm. The median (IQR) for O_3_ was 24.34 (22.88–26.23), 26.19 (24.76–27.29), and 28.01 (26.69–28.70) ppb. The median (IQR) for NO_2_ was 12.49 (10.46–13.62), 12.72 (10.58–14.15), and 12.91 (10.31–15.60) ppb. Exposure levels at patient locations are shown in Figure 1.

### Comparison of Exposure Model Performance

**Figure 2:**
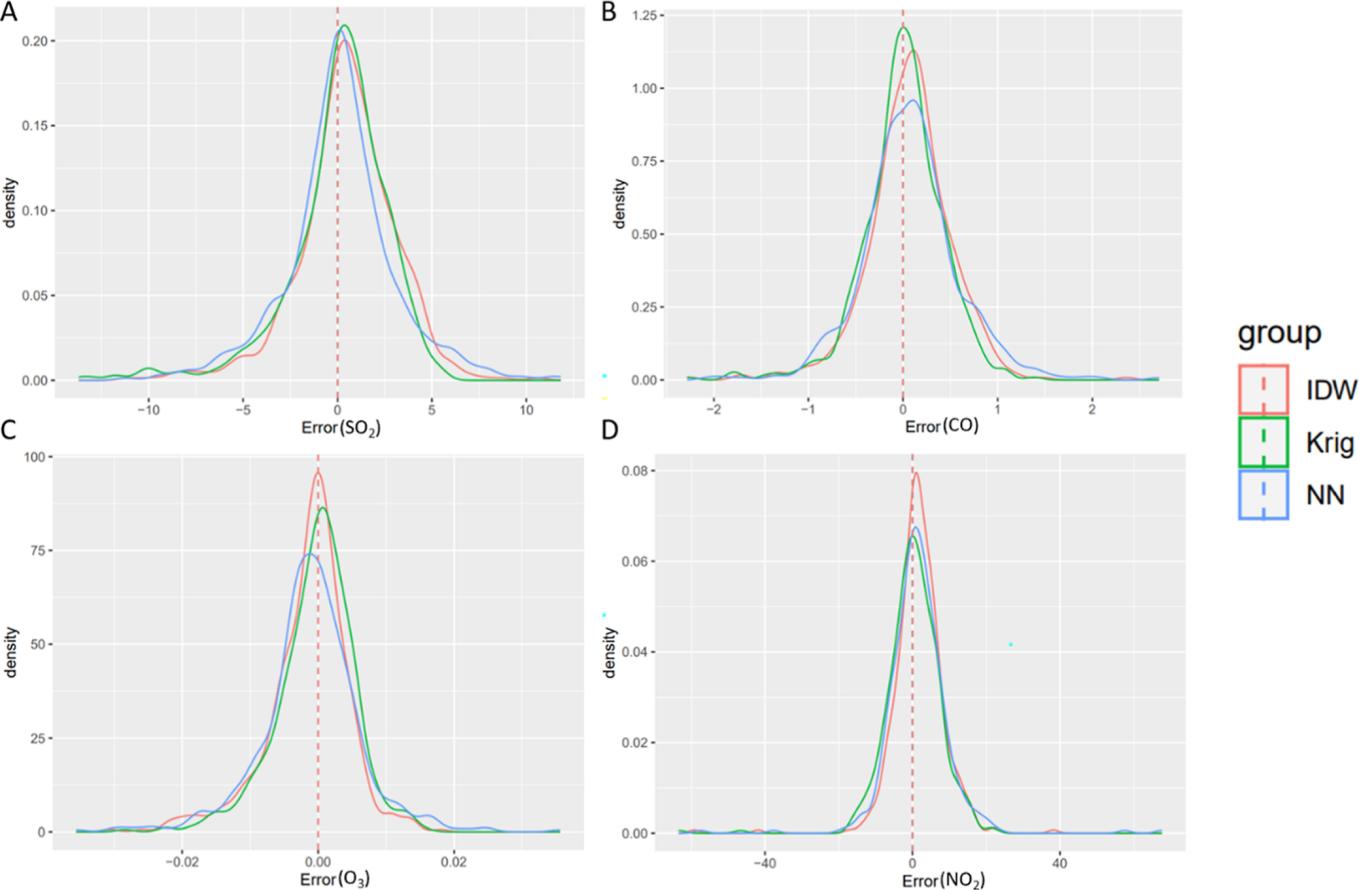
Distribution of spatial prediction errors (predicted value minus observed value) at the monitor locations for SO_2_ (A), CO (B), O_3_ (C), and NO_2_ (D) using the three modeling approaches in Jan 2000.

The cross-validated RMSE in Jan 2000 for SO_2_, CO, O_3_, and NO_2_ with three models is presented in Figure 2. Kriging had the lowest RMSE for CO [0.43 ppm (11%)], O_3_ [5.9 ppb (14%)], and NO_2_ [7.6 ppb (9%)]. Kriging and IDW had similar spatial RMSE values for SO_2_ (Tables 2 and S2).

**Table 2:** Spatial RMSE in Jan 2000 for SO_2_, CO, O_3_, and NO_2_ at the Monitor Locations^a^

	NN	IDW	Kriging
SO_2_ (ppb) (*n* = 583)	3.0 (13%)	2.6 (11%)	2.7 (12%)
CO (ppm) (*n* = 501)	0.52 (13%)	0.46 (12%)	0.43 (11%)
O_3_ (ppb) (*n* = 560)	7.0 (18%)	6.1 (15%)	5.9 (14%)
NO_2_ (ppb) (*n* = 401)	9.3 (12%)	8.4 (10%)	7.6 (9%)

a Numbers are absolute (proportional) RMSE. Absolute RMSE was calculated as RMSE = √(∑(e_î2)/n), where e_i = *y*_i - ŷ_i, which was then normalized with the range of each pollutant to calculate the proportional RMSE.

**Figure 3:**
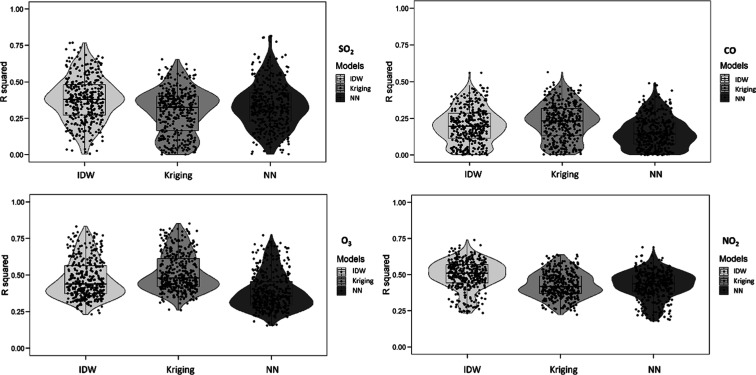
Spatial R^2^ distribution throughout all months for three methods and four pollutants.

The distribution of spatial R^2^ across all months is illustrated in Figure 3. The highest R^2^ was seen for IDW for SO_2_, followed by those for NN and Kriging. For CO and O_3_, Kriging was superior to IDW, with NN demonstrating the lowest R^2^. In the case of NO_2_, IDW exhibited better performance compared to Kriging, while NN showed the lowest R^2^.

**Figure 4:**
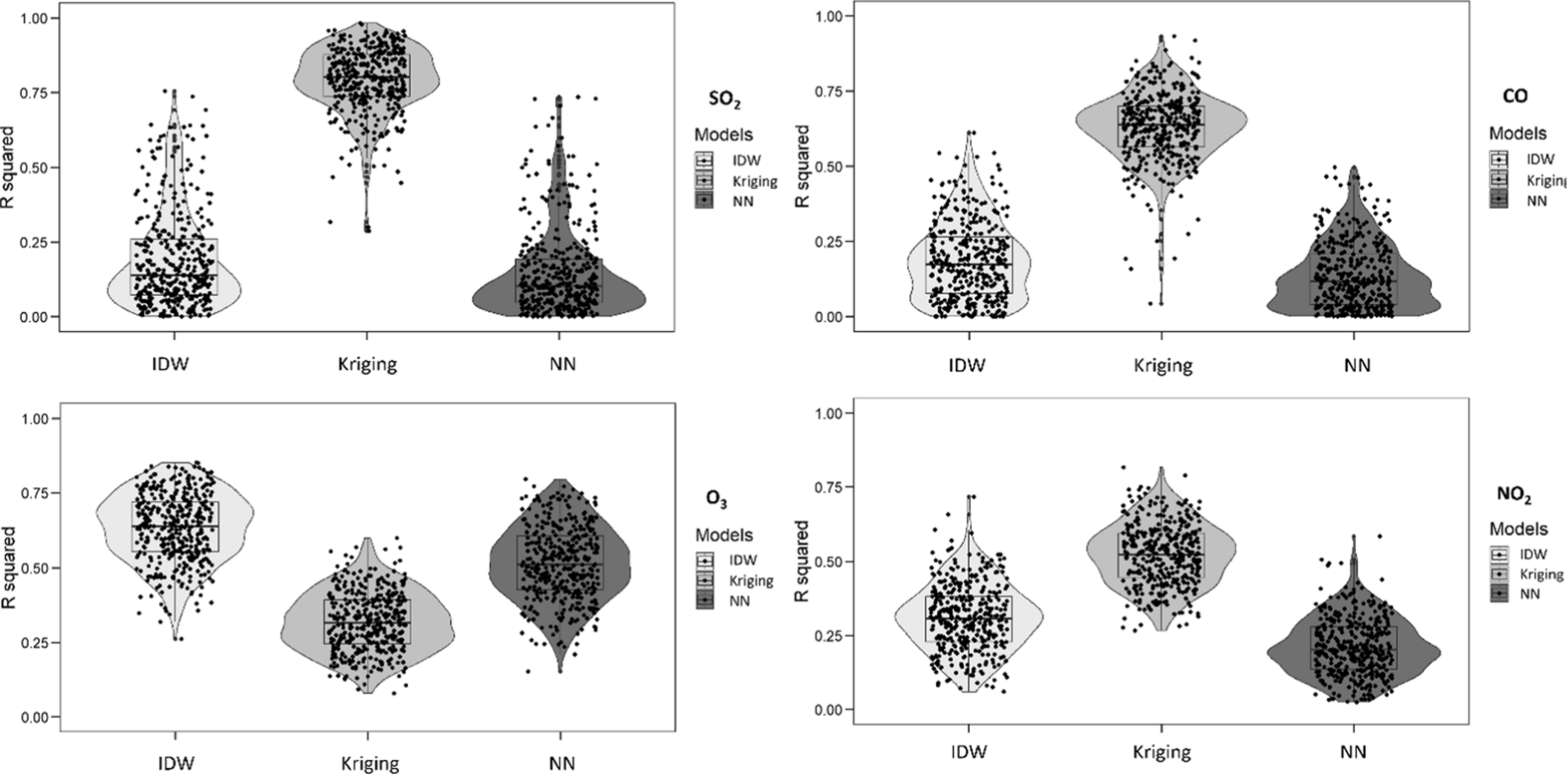
Temporal R^2^ distribution throughout all months for three methods and four pollutants.

Similarly, Figure 4 presents the distribution of the temporal R^2^ values for all monitors. For SO_2_ and NO_2_, Kriging had substantial advantages over IDW and NN in terms of R^2^. For CO, IDW recorded the highest R^2^, followed by Kriging and then NN. Regarding O_3_, IDW exhibited the highest R^2^, succeeded by NN and then Kriging.

The percentage of unfit months (1990–2020) for different gaseous pollutants using the Kriging model is summarized in Figure S3. Kriging still outperformed IDW and NN despite the lack of convergence in some months using the ‘gstat’ package in R. Upon examining the variogram for the unfitted months, many exhibited well-fitted patterns (Figure S4). The 10-fold cross-validation at the monitor site also supported the temporal validity of the Kriging model in predicting gaseous pollutants (Figure 4) over the NN and IDW.

### Association of Yearly Average Gaseous Pollutant Exposures with Baseline FVC and DLCO

Linear regression was performed to measure the influence of gaseous pollutants estimated from three methods on race-neutral baseline FVC percent predicted, with adjustment for smoking history (Table 3 and Figure S5). Increased exposures to SO_2_, CO, and NO_2_ were associated with a lower baseline FVC in all three methods. For CO and NO_2_, pollutant exposures estimated by Kriging demonstrated the strongest association with baseline FVC, followed by IDW and NN (e.g., NN β = −0.56 (CI: −0.76 to −0.36); IDW β = −0.77 (CI: −1.04 to −0.50), *p* = 0.001; Kriging β = −0.95 (CI: −1.28 to −0.61), all *p* < 0.001 for NO_2_). For SO_2_, pollutant estimates by Kriging and IDW demonstrated similar effect sizes, both outperforming NN. O_3_ by Kriging (β = 1.03 (CI: 0.36–1.72), *p* = 0.01) and IDW was associated with higher FVC (β = 0.81 (CI: 0.2–1.43), *p* = 0.03). Unadjusted models and models with both smoking history and ILD type (IPF absence/presence) demonstrated similar findings (Tables S3 and S4).

**Table 3:** Association of Gaseous Pollutants with Baseline Lung Function in Patients with fILD Based on Different Spatial Analysis Approaches after Adjusting for Smoking History^a^

	NN	IDW	Kriging			
	beta (95% CI)	*P* value	beta (95% CI)	*P* value	beta (95% CI)	*P* value
SO_2_–beta (95% CI, *P* value)						
DLCO *N* = 1030	0.07 (−0.05–0.18)	0.35	–0.06 (−0.15–0.04, 0.67)	0.33	–0.06 (−0.16–0.03)	0.26
FVC *N* = 1106	–1.09 (−1.64- −0.55)	<0.001	–1.16 (−1.61- −0.71)	<0.001	–1.13 (−1.57- −0.69)	<0.001
CO–beta (95% CI, *P* value)						
DLCO *N* = 1030	–1.86 (−2.79- −0.93)	0.001	–3.53 (−5.48- −1.58)	0.003	–3.88 (−6.31- −1.46)	0.008
FVC *N* = 1106	–11.69 (−16.02- −7.37)	<0.001	–25.32 (−34.39- −16.24)	<0.001	–30.73 (−41.99- −19.46)	<0.001
O_3_–beta (95% CI, *P* value)						
DLCO *N* = 1030	–0.06 (−0.22- −0.006)	0.03	0.001 (−0.13–0.13)	0.99	0.04 (−0.11–0.18)	0.68
FVC *N* = 1106	–0.01 (−0.47–0.44)	0.96	0.81 (0.20–1.43)	0.03	1.03 (0.36–1.72)	0.01
NO_2_–beta (95% CI, *P* value)						
DLCO *N* = 1030	–0.07 (−0.12- −0.03)	0.004	–0.08 (−0.15- −0.03)	0.01	–0.09 (−0.17- −0.02)	0.03
FVC *N* = 1106	–0.56 (−0.76- −0.36)	<0.001	–0.77 (−1.04- −0.50)	<0.001	–0.95 (−1.28- −0.61)	<0.001

a Change % predicted FVC or DLCO per one ppm increase in CO or one ppb increase in SO_2_, CO, and NO_2_ one year before baseline.

For DLCO, CO and NO_2_ levels derived from all three methods were associated with lower baseline DLCO. To ensure the robustness of using a one-year average, we conducted sensitivity analyses with time windows ranging from 1 month to 24 months. The standardized coefficients generally remained consistent, reflecting the same trend without significant fluctuations across the different time windows except for that of O_3_ (Figures S6−S8).

## Discussion

This study assesses three spatial analysis methods (IDW, NN, and Kriging) for estimating major gaseous pollutants (SO_2_, CO, O_3_, and NO_2_) in a cohort of patients with fILD. We found that the Kriging method is more accurate in exposure assignment, demonstrating robust performance across temporal and spatial variations. Furthermore, we found that pollutant exposures estimated by Kriging exhibited the strongest associations with fILD severity at diagnosis, as indicated by baseline FVC and DLCO. Specifically, CO showed significant associations with lower DLCO and FVC, where SO_2_ and NO_2_ were associated with lower FVC.

Ambient air pollutant exposure in environmental epidemiology often relies on measurement from sparsely distributed networks of regulatory ground-level pollutant monitors. The application of exposure data to clinical cohorts for epidemiologic research is limited by the reliability of estimates at patient locations and the ease of exposure assignment. Numerous studies have compared techniques for measuring air pollution exposure.^36,37^ However, these studies often focus on a single pollutant and lack a comprehensive assessment of the temporal and spatial validity. These limit their application in clinical studies. Therefore, there is a pressing need for an accessible and reliable model that provides accurate exposure estimation across various times and geographical locations.

The Simmons cohort of patients with fILD represents an ideal population to compare different exposure-matching methodologies. These patients are often older, making direct measurements logistically difficult and cost-prohibitive. Patients with fILD are vulnerable to the harmful impacts of ambient pollution.^38^ Even small inaccuracies in exposure estimation could attenuate or obscure exposure–response relationships due to the disease’s heterogeneous progression and sensitivity to environmental factors. Clinical data can be leveraged to compare different exposure-matching methodologies. The study participants in this cohort were recruited from highly urbanized and rural areas in the Northeastern US, reflecting regions impacted by heavy industrialization and traffic-related pollution. Steel mills and manufacturing plants have long been significant sources of gaseous pollutants, including sulfur dioxide (SO_2_) and nitrogen oxides (NO_*x*_).^39^ Areas characterized by high population density and heavy traffic experience elevated levels of gaseous pollutants such as carbon monoxide (CO) and NO_*x*_, primarily due to vehicular emissions. Furthermore, the Appalachian Mountains in the Northeastern region have distinctive topography, defined by its hilly terrain and valleys, which can lead to the entrapment of pollutants, particularly during temperature inversions.^40^ This region is influenced by coal-burning power plants. While these impacts may currently be less, they were likely greater in the earlier days of the Simmons Registry, when some patients in this study were recruited. The U.S. also witnesses variations in gaseous pollutants over time due to seasonal changes, such as fluctuations in temperature and precipitation.^41^ These temporal and geographic variations present challenges in developing models that accurately capture the spatial and temporal dynamics of major gaseous pollutants in the study area. In the pursuit of an effective model that captures spatial variations, various studies have been conducted to estimate gaseous pollutants. Berman et al. found that geospatial methods such as Kriging outperformed land use regression and IDW in predicting O_3_.^36^ Similarly, Laina et al. achieved comparable results in estimating NO_2_.^37^ A study conducted in South Korea reported that the Kriging method yielded more accurate and less biased results than other interpolation methods (NN and IDW), for pollutants such as PM_10_, SO_2_, NO_2_, O_3_, and CO.^42^ Another comprehensive study concluded that the Kriging method was suitable for estimating NO_2_, PM_10_, and O_3_ but not SO_2_ and CO.^43^ The temporal validity of the model can sometimes be more important. Policies influencing emissions from industrial or transportation sources can impact long-term trends in pollutant concentrations. Additionally, meteorological conditions, traffic patterns, and economic activities contribute to significant short-term fluctuations in the concentration. Therefore, achieving an accurate exposure assessment over time is crucial for estimating and inferring pollutant–outcome relationships. In our study, the Kriging method exhibited a more robust temporal and spatial relationship than the NN method with the reference measurements on EPA monitors. This underscores that advanced stochastic methods to model outdoor air pollution provide enhanced temporospatial resolution for exposure measurement. The strengths of these relationships were further confirmed through cross-validation models. Little is known about the properties of different interpolation methods for health effect estimation. Kim et al. reported that Kriging estimated PM_2.5_ exposures more accurately than neural networks. The overall health effect estimates for cardiovascular events were better evaluated with Kriged exposure, particularly when comparing results based on coverage probability.^44^ Son et al. compared air pollution estimated from Kriging, nearest neighbor, IDW, and average across all monitors with lung function and found only Kriging estimation had a significant clinical association.^37^

O_3_ estimated by IDW and Kriging was associated with increased lung function. As an extremely reactive chemical species,^45^ ground-level ozone formation is influenced by atmospheric chemical processes and meteorological factors, which complicates its measurement using spatial models.^46,47^ Ozone also exhibits significant seasonal variations, typically peaking in the late summer and early fall, as confirmed by our sensitivity test comparing different exposure windows. While all other gaseous pollutants showed consistent standardized coefficients, ozone behaved differently, suggesting that it may not be suitable for estimation by ground-based monitors. On the other hand, we found that NO_2_ was associated with lowered lung function and gas transfer ability. Exposure to NO_2_ has been shown to induce inflammation in the airways, which increases airway responsiveness to irritants and can reduce lung function by causing bronchoconstriction.^48,49^ NO_2_ is a reactive free radical that can cause peroxidation of membrane lipids, leading to biochemical and metabolic abnormalities in lung endothelial cells.^49^ SO_2_ has similar toxicity mechanism as NO_2_, but it only showed negative effect on lung function in our study. We found that CO has a negative effect on lung function and the gas transfer ability. Ambient carbon monoxide (CO) exposure has been associated with adverse respiratory health outcomes, including increased risk of hospitalization and outpatient visits for various lung diseases, including asthma, COPD, and IPF.^50−52^ Low-dose CO exposure influenced the expression of oxidative phosphorylation-related genes in IPF patients.^53^ The potential mechanism might include oxidative stress and epigenetic changes through upregulation of aberrant inflammatory or profibrotic responses.^54,55^ Furthermore, associations were observed at exposure levels significantly lower than the EPA recommended thresholds, highlighting the detrimental impact of gaseous pollutants on the respiratory system, particularly in high-risk populations of patients with fILD.

While this work provides valuable information, demonstrating how Kriging outperforms IDW and NN in exposure prediction and clinical outcome evaluation, there remain limitations to this study. The absence of detailed data on patient absences, such as hospital admissions, introduces the potential for exposure misclassification. However, we acknowledge this limitation while noting that the participants in this study are generally less mobile. As a result, we postulate that any mobility-related bias is unlikely to significantly affect the overall findings. We did not adjust for short-term climatic changes, such as temperature or humidity, as they are unlikely to significantly impact the trajectory of a disease with such a long and chronic course. This was further supported by our sensitivity analysis, which examined time windows ranging from 1 month to 24 months and demonstrated consistent results, even without accounting for these acute variables. Besides, elements of Kriging models may not be applicable for different time points. By using single parameters for each pollutant, the program reported a lack of convergence for many months, indicating that the variogram parameters could not fit the variogram models in some iterations.^56^ To the best of our knowledge, this reflects the first study to comprehensively describe the temporal limitations of Kriging in estimating gaseous pollutants. Possible reasons for this inadequacy include insufficient data, violations of stationarity assumptions, and misspecification of variogram models.^57,58^ Kriging assumes spatial structure constancy across the domain; however, the mean values of the gaseous pollutants declined over long periods. Additionally, seasonal variations and differences in monitor availability further influenced model fitting. Despite these challenges, many variograms for the problematic months exhibited well-fitted patterns. This may be explained by model sensitivity or iteration. The problem can potentially be alleviated by modifying the function to make it faster and less sensitive but potentially less accurate.^56^

This study is among the first to directly compare the performance of three spatial interpolation techniques (NN, IDW, and Kriging) in the context of gaseous pollutant exposure estimation for fILD patients. While IDW and NN are widely applied in epidemiological studies due to their simplicity, Kriging remains underutilized, despite its potential for higher accuracy. The superior performance of the Kriging method, as indicated by lower prediction error and higher spatial and temporal R^2^, has critical implications for minimizing exposure misclassification (Berkson error). Accurate pollutant exposure estimates are essential for understanding the environmental determinants of fILD progression and exacerbations. High spatial R^2^ ensures that local variations in exposure levels, which may disproportionately affect certain subpopulations, are captured with precision. Similarly, high temporal R^2^ allows for accurate modeling of short-term exposure peaks, which are particularly relevant for studying acute health impacts such as symptom acute exacerbation or hospitalizations. By improving the reliability of exposure estimates, Kriging enhances the ability to detect true exposure–response relationships, thereby supporting more robust conclusions about the health effects of pollutants in fILD patients. These advancements are essential for guiding targeted public health interventions and environmental regulations to protect this vulnerable population. This work provides critical insights that can improve epidemiological analyses and guide future applications of advanced spatial modeling techniques in rare disease research. Future research should focus on testing these methods using independent data sets or in diverse geographic regions to evaluate the robustness and reliability of pollutant exposure estimates across varying environmental contexts. Such efforts would help establish the broader applicability of our findings and confirm the utility of these methods for exposure assessment in diverse settings.

In conclusion, this research offers a simple and reliable approach for estimating levels of air pollutants (CO, NO_2_, O_3_, and SO_2_) from monitoring stations, which can be applied to epidemiologic studies. By comprehensively evaluating three gaseous pollutant interpolation methods (nearest neighbor, inverse distance weighting, and Kriging), it was determined that Kriging had the highest validity in estimating pollutant exposures at locations with complex geographical conditions and temporal fluctuations. These interpolations were applied to a cohort of patients with fibrotic interstitial lung disease (fILD), identifying adverse associations of CO with lung gas transfer ability and NO_2_ and SO_2_ lowering lung function, especially when exposures were estimated using a Kriging approach. This study provides valuable insights for future health studies to assess exposure to air pollutants, which may be leveraged to advocate for environmental health policies aimed at protecting vulnerable populations, including individuals with fILD.

## Supporting Information Available

Parameters used in the Kriging model for each pollutant; numeric summary of error distribution in model cross-validation; association between gaseous pollutants and baseline lung function without adjusting for covariates; adjustments for smoking history and IPF (absence/presence); gaseous pollutants at patients’ locations; EPA ground monitor locations for gaseous pollutants; annual percentage of unfitted months for gaseous pollutants using the Kriging model; variograms of the unfitted months; scatter plots depicting associations between DLCO, FVC, and 1 year average pollution levels; and sensitivity analyses on the association (standardized coefficient) between pollution at different exposure time windows and DLCO or FVC (PDF)

## Notes

This was an independent, investigator-initiated study supported by the Boehringer Ingelheim (BI). BI had no role in the design, analysis, or interpretation of the results in this study. GCG has received research funding and support through the PFF Scholars Award Program, the University of British Columbia Clinician Investigator Program and Division of Respiratory Medicine, a Boehringer Ingelheim Investigator Initiated Study grant, the Canadian Institutes of Health Research, the Canadian Lung Association, Michael Smith Health Research BC. She has received speaking honoraria from Boehringer Ingelheim within the past year. DJK is supported in part by AR060780, HL133232, UL1 TR001857, AR076024 NIH grants, and Boehringer-Ingelheim grants and receives collaborative research funding from Regeneron Pharmaceuticals, outside of the submitted work. SMN has received funding from a Boehringer Ingelheim Investigator Initiated Study grant and the Division of Pulmonary, Allergy, Critical Care, and Sleep Medicine at the University of Pittsburgh. JPF has received funding from the Heinz Endowments and U.S. Dept. of Housing and Urban Development and is supported in part by ES036033, ES036033, and ES034701 NIH grants.

## Abbreviations

fILD: fibrotic interstitial lung disease
IPF: idiopathic pulmonary fibrosis
NN: nearest neighbor
IDW: inversed distance weighting
PM_2.5_: particulate matter ≤2.5 μm in diameter
CO: carbon monoxide
NO_2_: nitrogen dioxide
O_3_: ozone
SO_2_: sulfur dioxide
RMSE: root-mean-square error
FVC %: percent predicted forced vital capacity
DLCO: diffusion capacity of the lung for carbon monoxide
ppm: parts per million
ppb: parts per billion
