# Supplementary material for: Gaseous Air Pollutants and Lung Function in Fibrotic Interstitial Lung Disease (fILD): Evaluation of Different Spatial Analysis Approaches
Source: Environ Sci Technol. 2025 Mar 22;59(12):5936–45. doi: 10.1021/acs.est.4c11275 (PMC11966764; doi:10.1021/acs.est.4c11275)

Supporting Information

for

Gaseous air pollutants and lung function in fibrotic interstitial lung disease (fILD): Evaluation of different spatial analysis approaches

Shuangjia Xue[1], Matthew J Broerman[2], Gillian C Goobie[2,3,4], Daniel J Kass [2,5], James P Fabisiak[1], Sally E Wenzel[1], and Seyed Mehdi Nouraie [1,2,5*]

1. Department of Environmental and Occupational Health, Graduate School of Public Health, University of Pittsburgh, Pittsburgh, Pennsylvania, 15261, U.S.A.
2. Division of Pulmonary, Allergy, Critical Care, and Sleep Medicine, Department of Medicine, University of Pittsburgh, Pittsburgh, Pennsylvania, 15213 U.S.A.
3. Division of Respiratory Medicine, Department of Medicine, University of British Columbia, Vancouver, British Columbia, V5Z 1M9, Canada
4. Centre for Heart Lung Innovation, St. Paul's Hospital, University of British Columbia, Vancouver, British Columbia, V5Z 1M9, Canada
5. Simmons Center for Interstitial Lung Disease, Division of Pulmonary, Allergy and Critical Care Medicine, Department of Medicine, University of Pittsburgh, Pittsburgh, Pennsylvania, 15213, U.S.A.

Correspondence to:

Seyed Mehdi Nouraie, MD, PhD

Division of Pulmonary, Allergy, Critical Care, and Sleep Medicine, Department of Medicine, University of Pittsburgh, Pittsburgh, Pennsylvania, 15213, U.S.A.

nouraies@upmc.edu

Summary: 10 pages, 4 Tables, 8 Figures.

Table S1 presents parameters used in the kriging model for each pollutant. Table S2 provides a numeric summary of error distribution in model cross-validation. Table S3 shows the association between gaseous pollutants and baseline lung function without adjusting for covariates, while Table S4 includes adjustments for smoking history and IPF (absence/presence). Figure S1 maps gaseous pollutants at patients' locations, and Figure S2 displays EPA ground monitor locations for gaseous pollutants. Figure S3 illustrates the annual percentage of unfitted months for gaseous pollutants using the kriging model, and Figure S4 presents variograms of the unfitted months. Figure S5 and Figure S6 show scatter plots depicting associations between DLCO, FVC, and 1-year average pollution levels. Figures S7 and S8 provide sensitivity analyses on the association (standardized coefficient) between pollution at different exposure time windows and DLCO or FVC.

Table S1. The nugget, range, and sill are used to fit a semivariogram model

Pollutants	Model	Psill	Nugget	Range
SO_2	Exp	400	200	10
CO	Sph	300000	200000	2
O_3	Exp	60	25	2
NO_2	Exp	100	25	5

The model type refers to the mathematical function used to fit the empirical semivariogram. The sill is the value that the semivariance reaches and levels off at. The nugget represents the semivariance at zero distance. The range is the distance at which the semivariance levels off and reaches a constant value

Table S2. Error distribution in cross-validation at monitor location for different gaseous pollutants presented as median (IQR) and standard error

Pollutants	NN	IDW	Kriging
2000.Jan			
SO_2	0.11 (-1.22, 1.57) 2.93	0.54 (-0.73, 2.04) 2.59	0.37 (-0.97, 1.64) 2.72
CO	0.07 (-0.20, 0.33) 0.52	0.08 (-0.16, 0.32) 0.45	0.03 (-0.20, 0.24) 0.43
O_3	-0.0009 (-0.004, 0.003) 0.007	-0.0005 (-0.004, 0.002) 0.006	0.0003 (-0.003 , 0.003) 0.006
NO_2	1.15 (-2.57, 5.37) 8.55	1.45 (-1.46, 5.30) 7.10	0.42 (-3.62, 4.75) 7.56
2017.Jan			
SO_2	0.02 (-0.47, 0.52) 2.84	0.22 (-0.27, 0.62) 2.78	0.28 (-0.31, 0.62) 3.24
CO	0.03 (-0.09, 0.13) 0.19	0.04 (-0.07, 0.11) 0.16	0.01 (-0.07, 0.09) 0.15
O_3	-0.0006 (-0.003, 0.002) 0.006	-0.0006 (-0.003, 0.002) 0.005	-0.0001 (-0.003, 0.002) 0.005
NO_2	1.13 (-3.28, 3.95) 5.50	1.74 (-1.85, 4.44) 4.57	0.38 (-2.74, 3.06) 4.99

The numeric summary of RMSE for three models in random months.

Table S3. Association between gaseous pollutants and baseline lung function in patients with fILD based on different spatial analysis approaches. (no adjustment for covariate)

**SO_2 – Beta (95% CI, P value)**			
	**NN**	**IDW**	**Kriging**
DLCO N = 1030	0.06 (-0.05- 0.17, 0.40)	0.01 (-0.10- 0.12, 0.85)	-0.05 (-0.14- 0.04, 0.35)
FVC N = 1106	-0.63 (-1.14- -0.12, 0.04)	-0.96 (-1.46- -0.45, 0.002)	-1.17 (-1.60- -0.73, <0.001)
**CO – Beta (95% CI, P value)**			
	**NN**	**IDW**	**Kriging**
DLCO N = 1030	-1.73 (-2.63- -0.83, 0.003)	-3.36 (-5.26- -1.45, 0.004)	-3.64 (-6.04- -1.25, 0.012)
FVC N = 1106	-12.53(-16.70- -8.36, <0.001)	-27.76 (-36.61- -18.92, <0.001)	-33.55 (-44.66- -22.44, <0.001)

O_3 – Beta (95% CI, P value)			
	**NN**	**IDW**	**Kriging**
DLCO N = 1030	-0.10 (-0.21- -0.002, 0.11)	-0.05 (-0.19- 0.08, 0.52)	-0.04 (-0.19- 0.11, 0.67)
FVC N = 1106	0.22 (-0.28- 0.71, 0.47)	0.59 (-0.05- 1.23, 0.13)	0.80 (0.08- 1.52, 0.07)
**NO_2 – Beta (95% CI, P value)**			
	**NN**	**IDW**	**Kriging**
DLCO N = 1030	-0.05 (-0.15- 0.06, 0.47)	-0.06 (-0.15- 0.04, 0.33)	-0.07 (-0.15- 0.004, 0.12)
FVC N = 1106	-1.20 (-1.68- -0.72, <0.001)	-1.17 (-1.61- -0.73, <0.001)	-1.05 (-1.41- -0.69, <0.001)

Table S4. Association between gaseous pollutants and baseline lung function in patients with fILD based on different spatial analysis approaches. (adjusted for smoking history and IPF absence/presence)

SO_2 – Beta (95% CI, P value)			
	**NN**	**IDW**	**Kriging**
DLCO N = 1030	0.11 (-0.009- 0.22, 0.13)	0.003 (-0.09- 0.10, 0.96)	-0.005 (-0.10- 0.09, 0.93)
FVC N = 1106	-1.35 (-2.29- -0.42, 0.003)	-1.00 (-1.45- -0.54, <0.001)	-0.97 (-1.41- -0.53, <0.001)
**CO – Beta (95% CI, P value)**			
	**NN**	**IDW**	**Kriging**
DLCO N = 1030	-1.35 (-2.29- -0.42, 0.02)	-2.53 (-4.48- -0.58, 0.03)	-2.76 (-5.18- -0.35, 0.06)
FVC N = 1106	-10.22(-14.59- -5.84, 0.001)	-22.41 (-31.57- -13.26, <0.001)	-27.47 (-38.80- -16.14, <0.001)
**O_3 – Beta (95% CI, P value)**			
	**NN**	**IDW**	**Kriging**
DLCO N = 1030	-0.08 (-0.18- 0.02, 0.18)	-0.06 (-0.20- 0.07, 0.42)	-0.04 (-0.19- 0.11, 0.67)
FVC N = 1106	-0.05 (-0.51- 0.40, 0.85)	0.62 (-0.003- 1.24, 0.10)	0.82 (0.13- 1.5, 0.05)
**NO_2 – Beta (95% CI, P value)**			
	**NN**	**IDW**	**Kriging**
DLCO N = 1030	-0.05 (-0.09- 0.03, 0.08)	-0.05 (-0.10- 0.01, 0.19)	-0.04 (-0.11- 0.03, 0.37)
FVC N = 1106	-0.49 (-0.69- -0.28, <0.001)	-0.68 (-0.95- -0.40, <0.001)	-0.83 (-1.17- -0.50, <0.001)

Figure S1. Levels of gaseous pollutants (12 months average before the date of enrollment) at patients' location based on IDW and NN

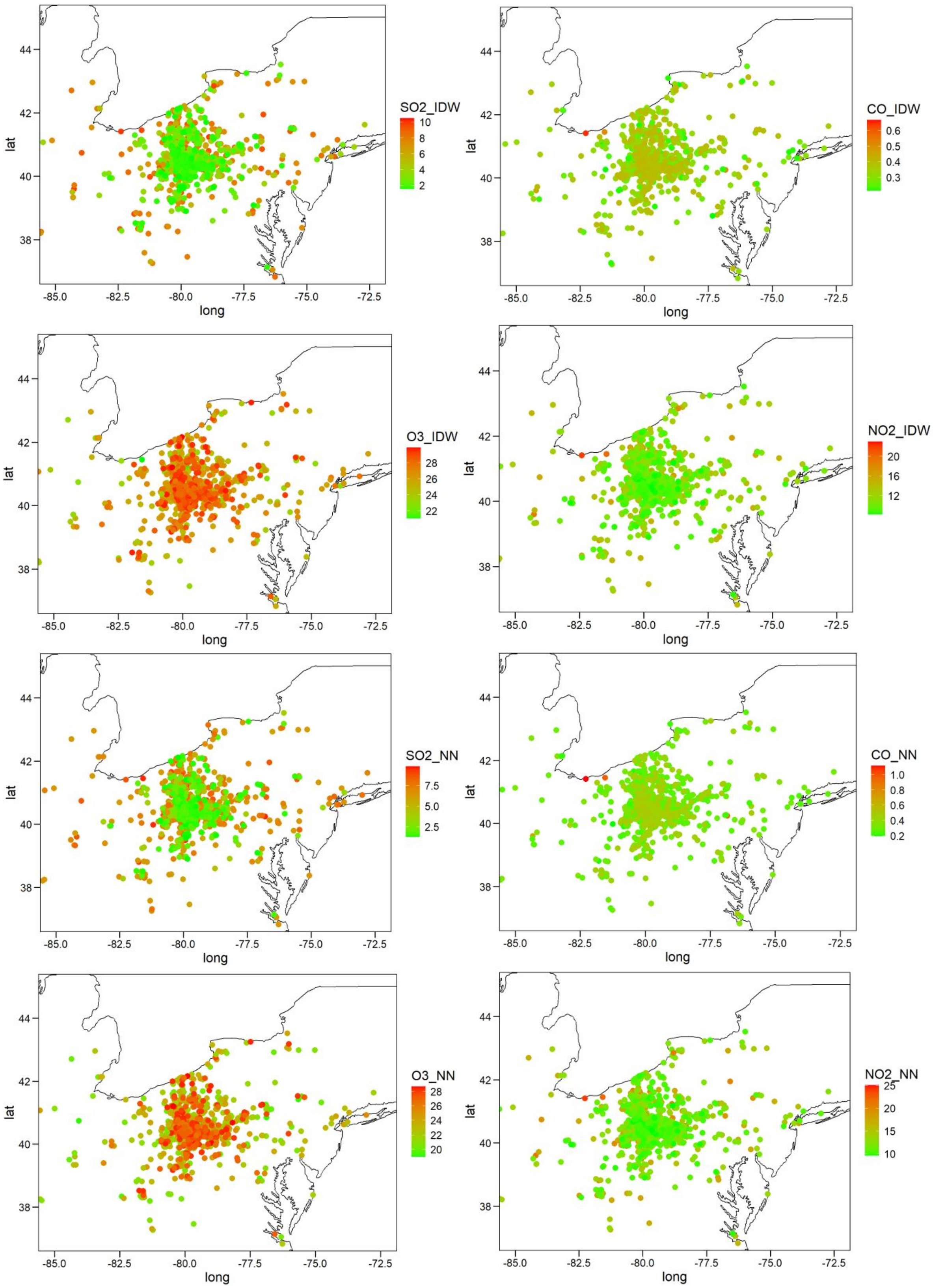

Unit: SO_2 ppb, CO ppm, O_3 ppb, NO_2 ppb

Figure S2. EPA ground monitor locations for gaseous pollutants

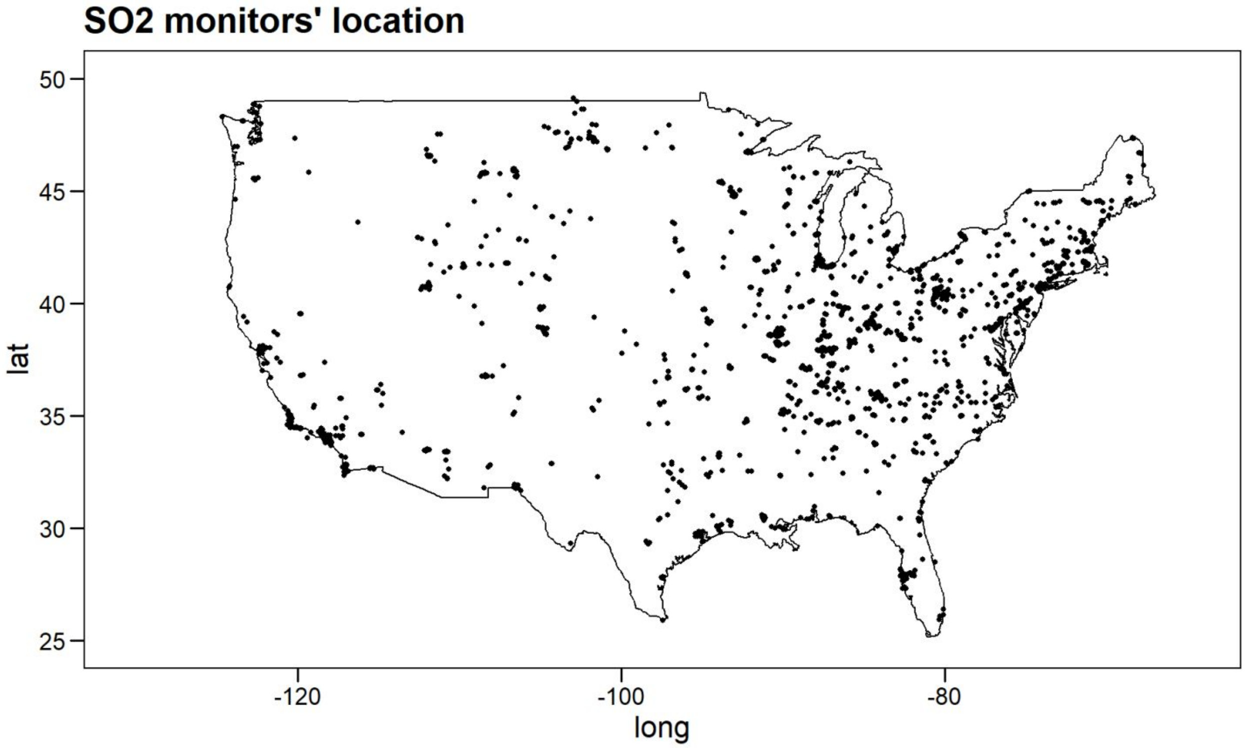

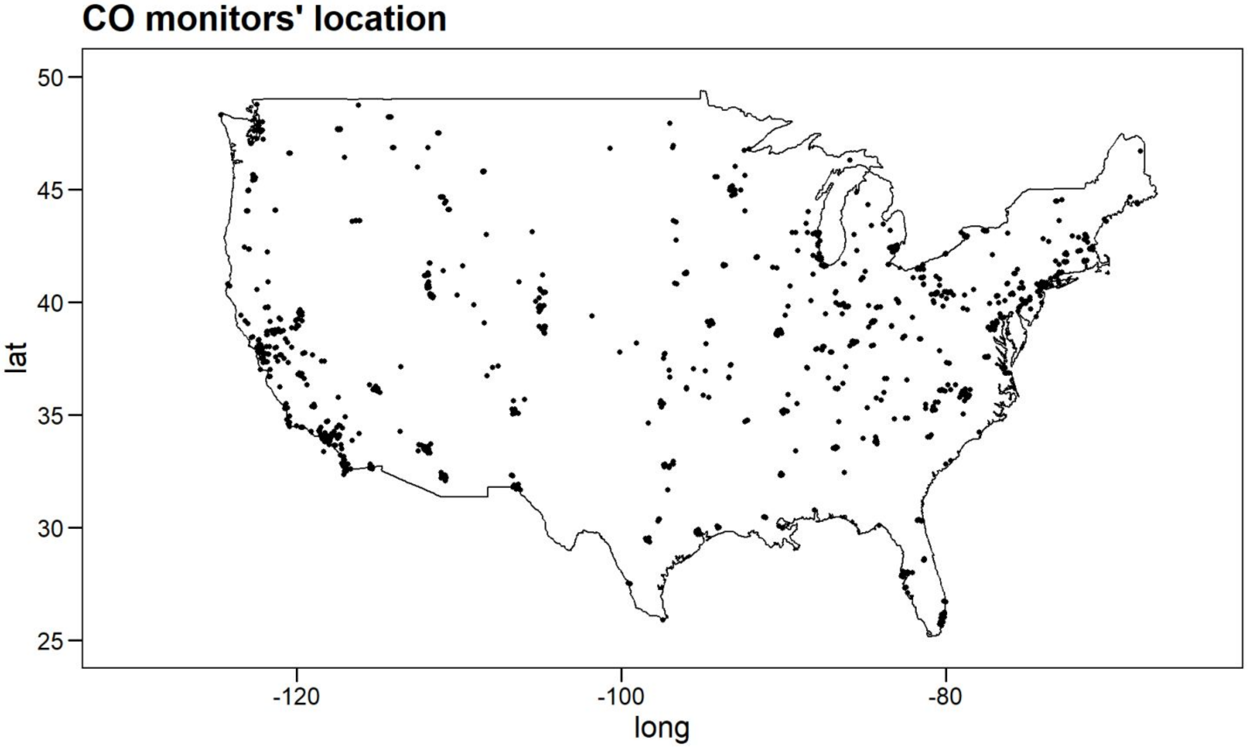

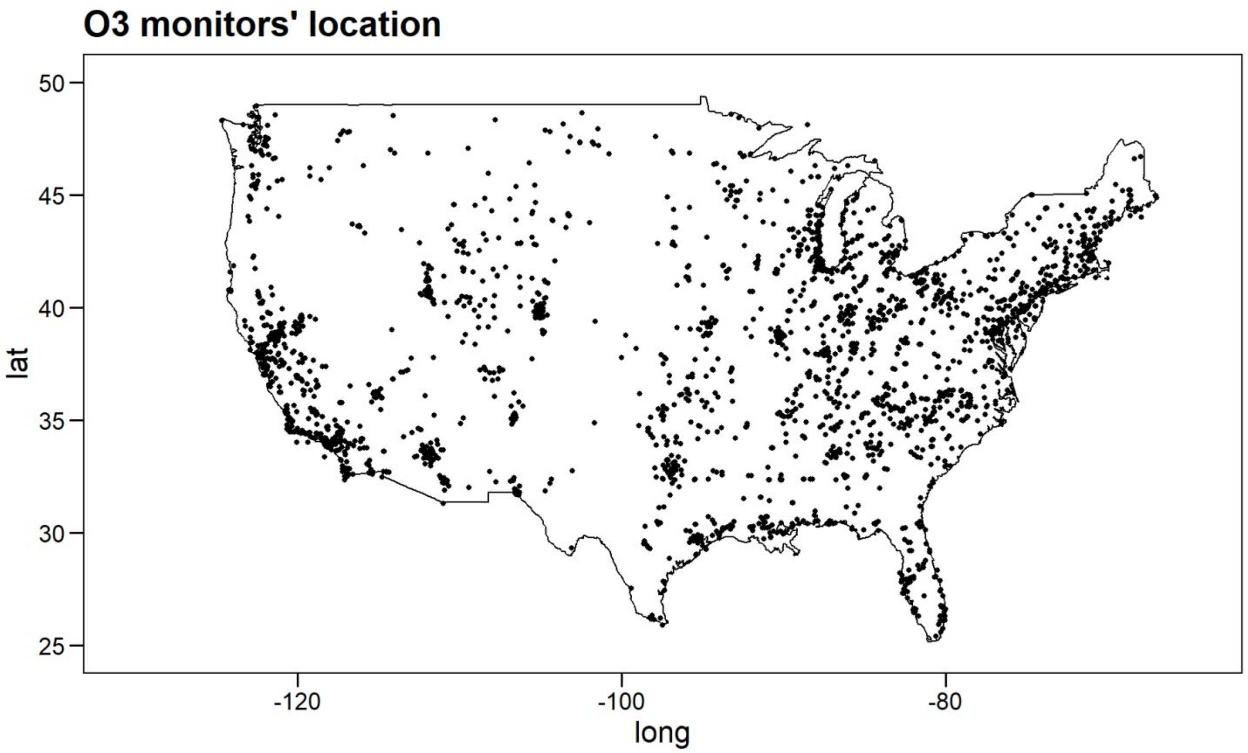

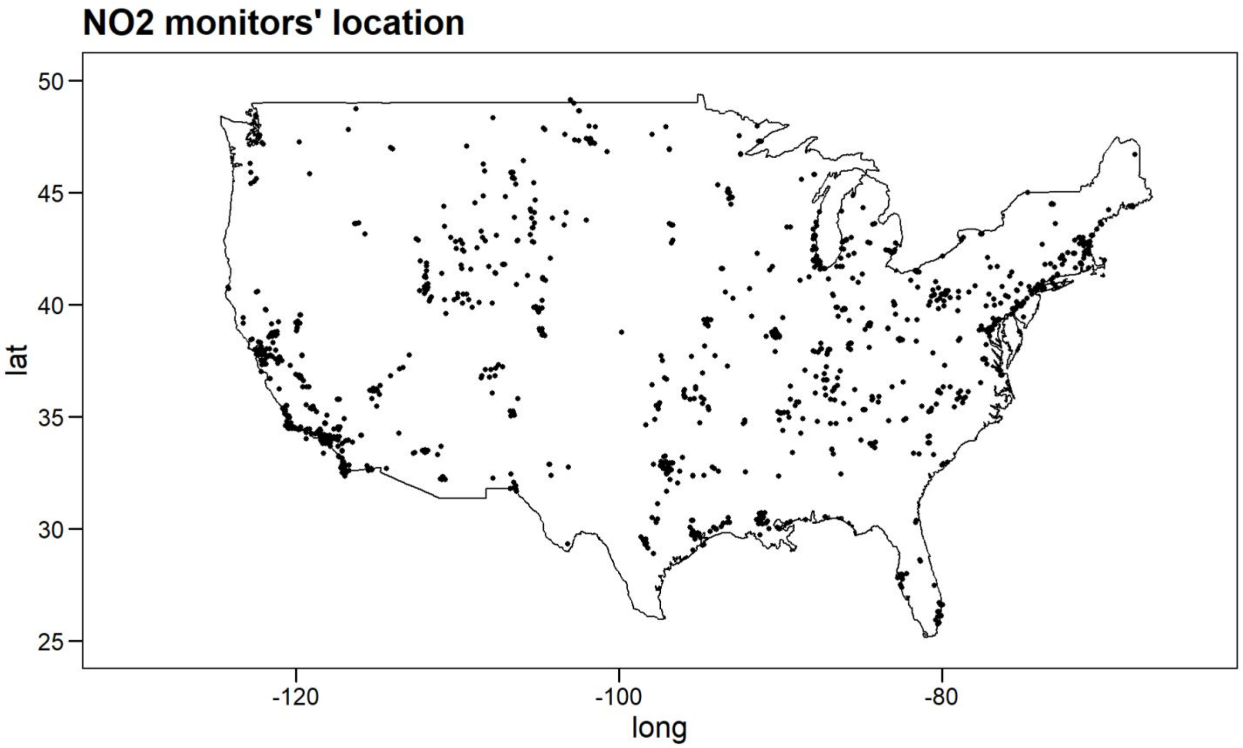

Figure S3. Annual percentage of unfitted months for gaseous pollutants

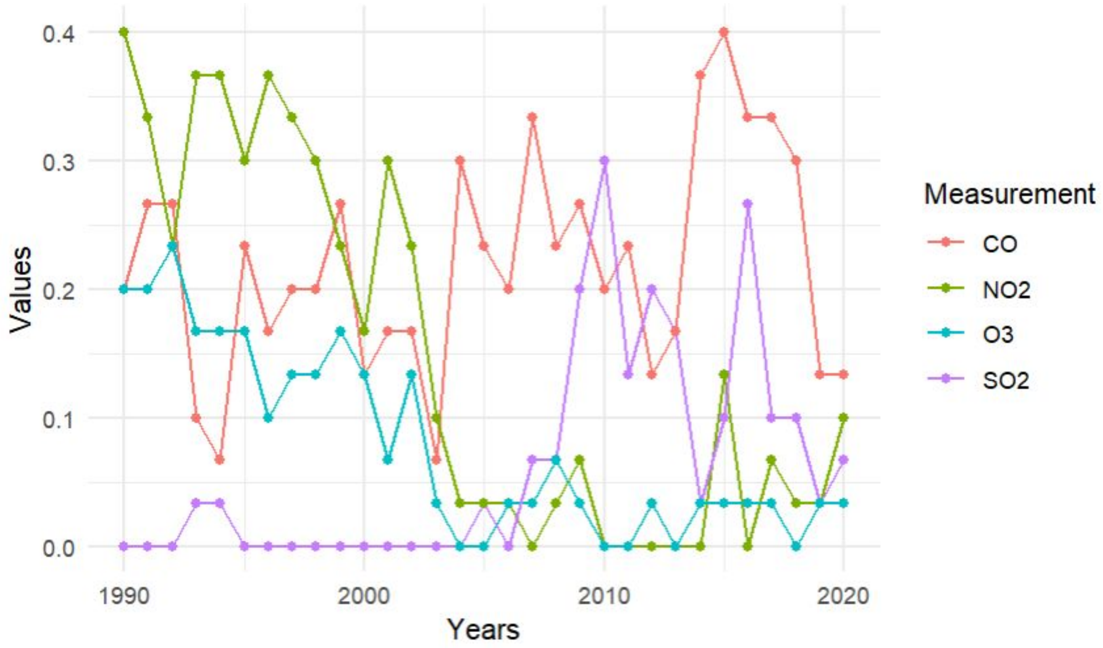

The percentage of months each year from 1990 to 2020 in which the Kriging model was reported as unfit for each gaseous pollutant.

Figure S4. Variograms of the unfitted month for gaseous pollutants (A. CO, B. NO2, C. O3, D. SO2)

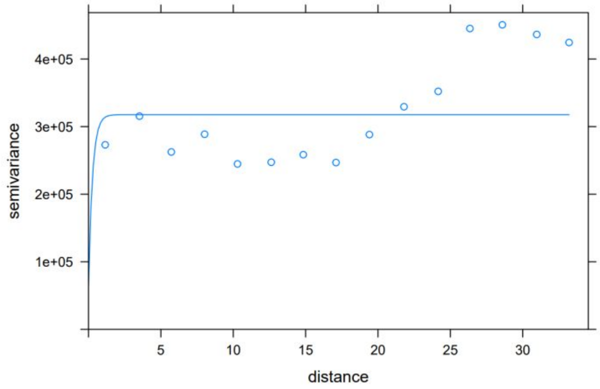

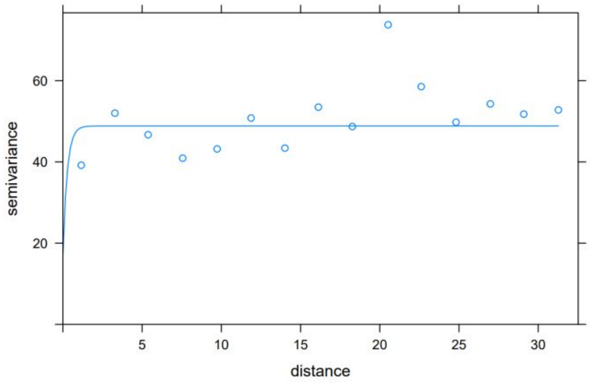

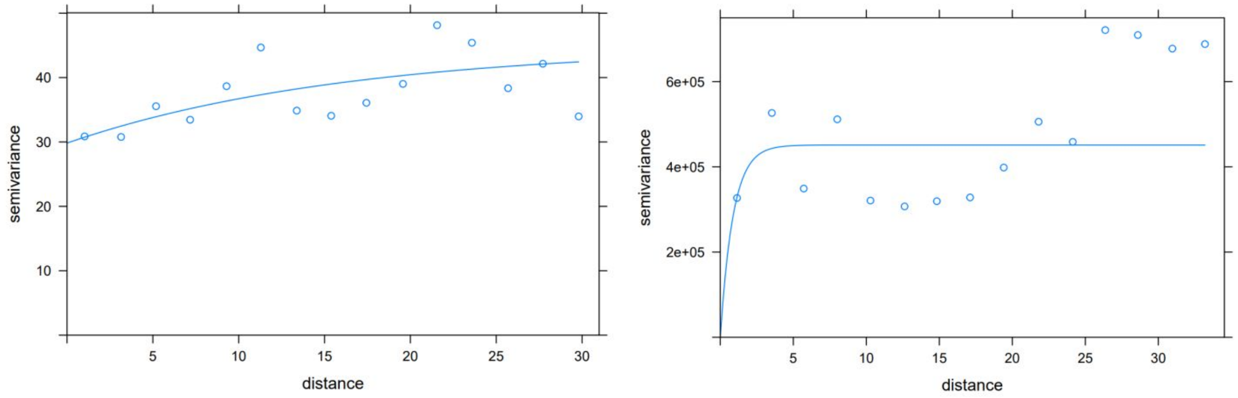

The variogram and Kriging model for a randomly selected month being reported as unfit for each pollutant.

Figure S5. Scatter plots showing the association between DLCO and 1-Year Average Pollution Levels

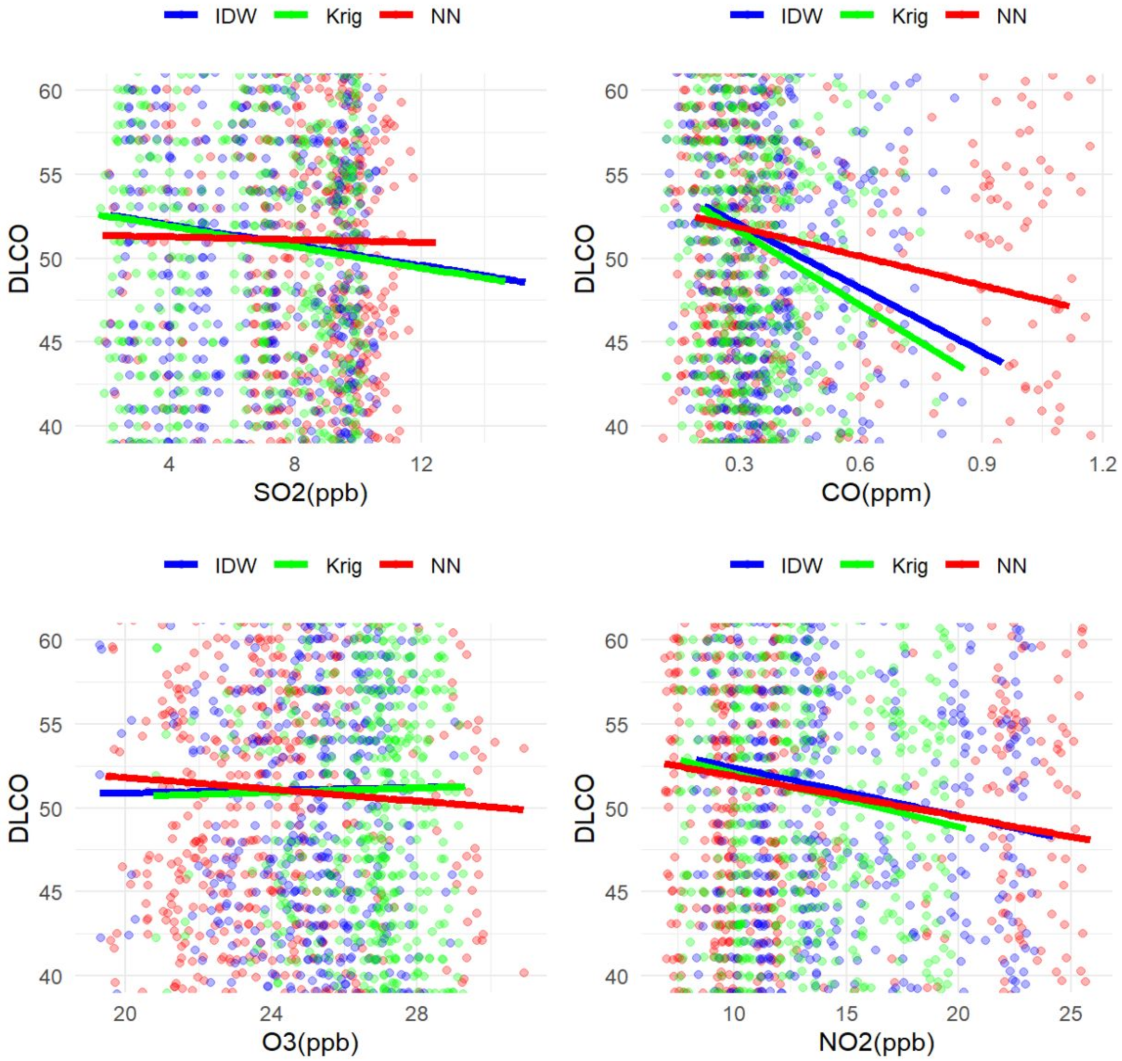

Figure S6. Scatter plots showing the association between FVC and 1-Year Average Pollution Levels

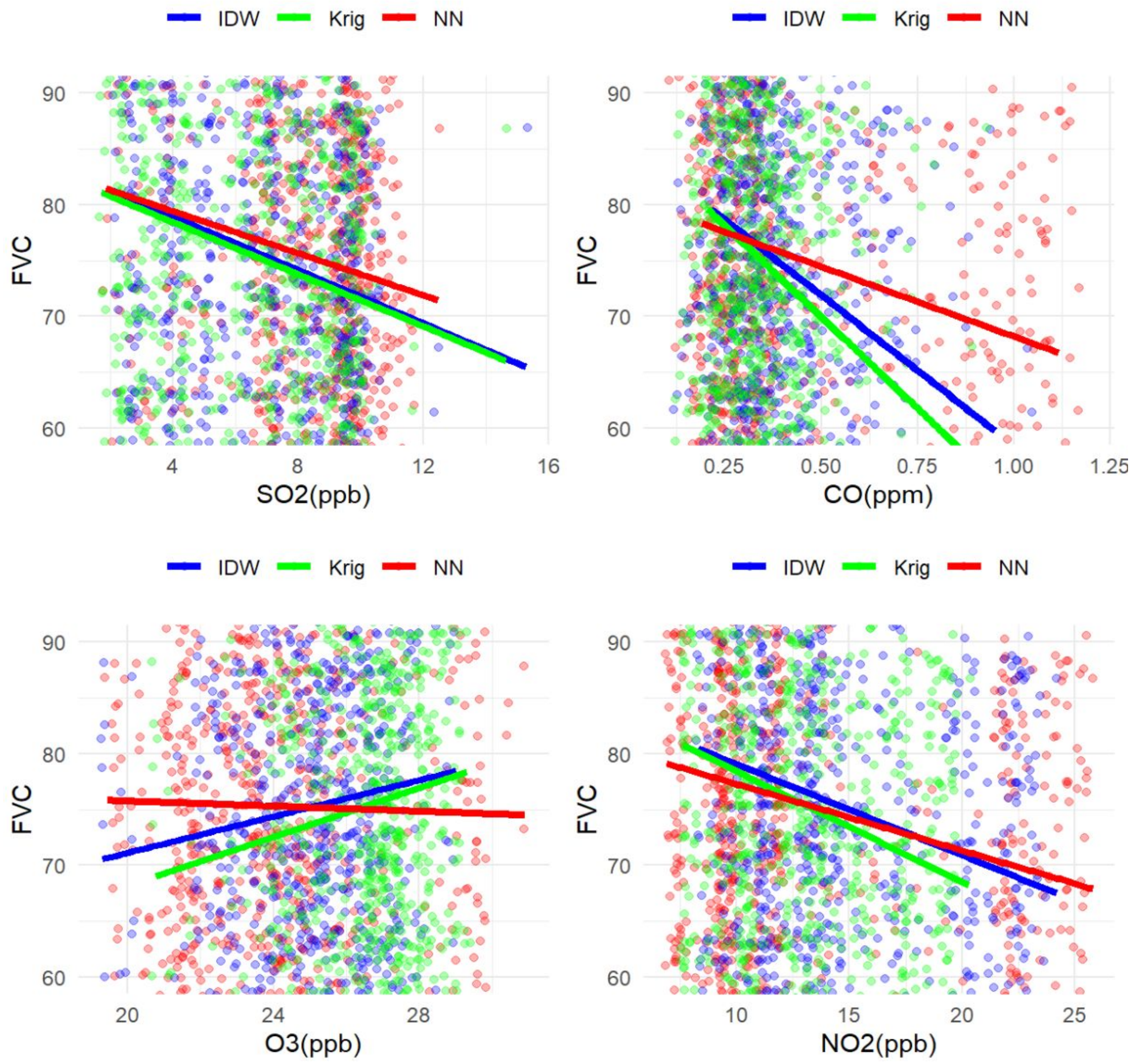

Figure S7. Sensitivity Analysis: Association (standardized coefficient) between pollution at different exposure time windows and DLCO

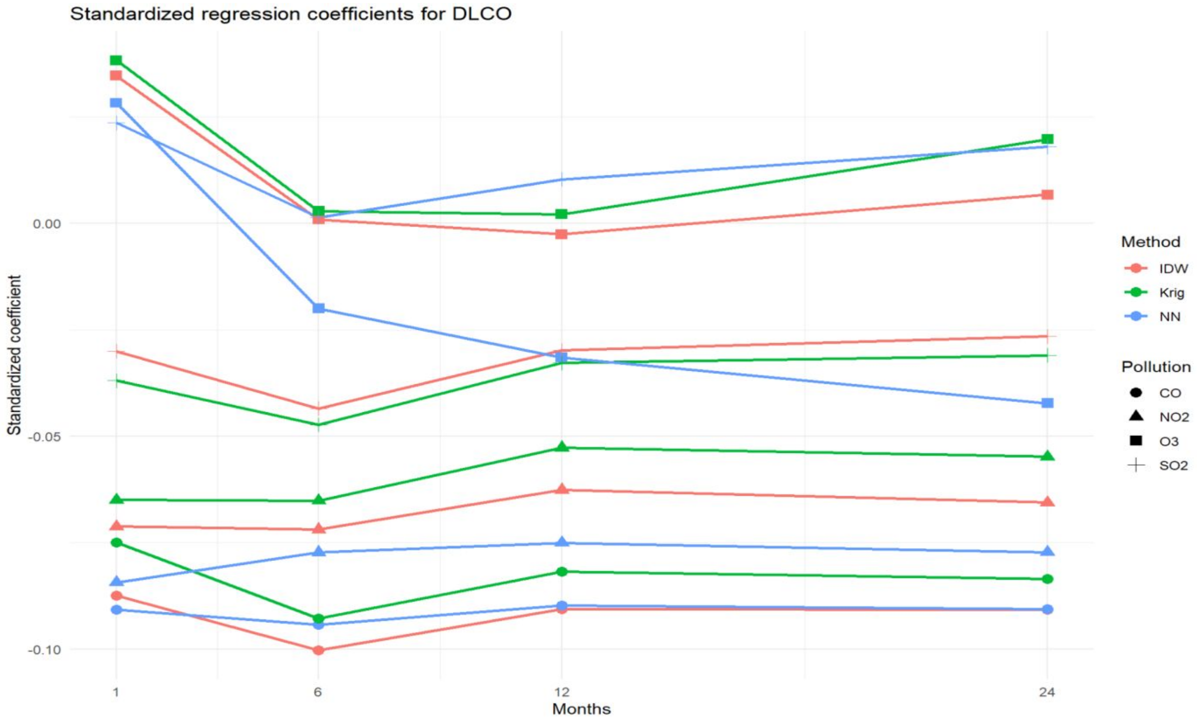

Figure S8. Sensitivity Analysis: Association (standardized coefficient) between pollution at different exposure time windows and FVC

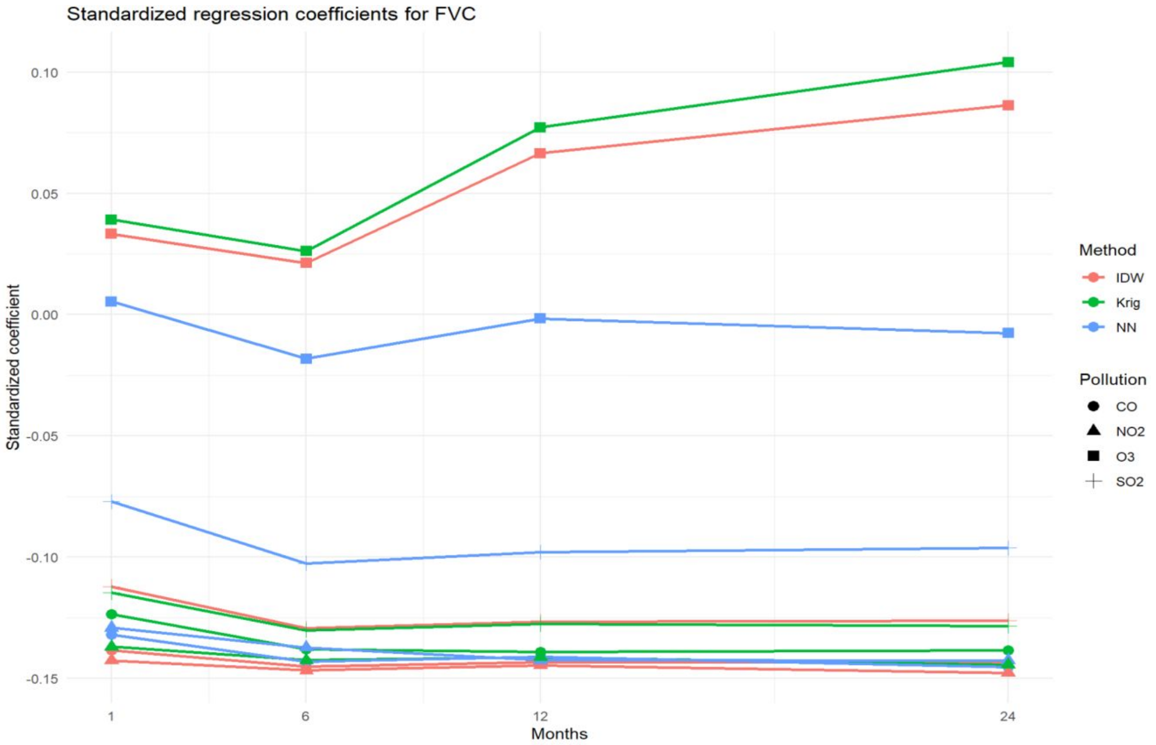

Supplement: Supplementary file 1 — es4c11275_si_001.pdf [file es4c11275_si_001.pdf]
